# Analysis of sit-ski alpine skiing trajectories based on an inverted pendulum model

**DOI:** 10.1038/s41598-025-30787-8

**Published:** 2025-12-04

**Authors:** Xu Zhiyi, Lu Jie, Xu Qinghua, Lin Mingjie, Liu Tao, Wang Xiangdong

**Affiliations:** 1 School of Physical Education, Jimei University, Xiamen, Fujian China; 2 China Administration of Sports for Persons with Disabilities, Beijing, China

**Keywords:** Sit-ski alpine skiing, Inverted pendulum model, Trajectory analysis, Center of mass trajectory, Gate-turning, Computational models, Computational biophysics

## Abstract

This study analyzes sit-ski alpine skiing trajectories during gate-turning phases using an inverted pendulum model combined with an advanced kinematic testing system involving inertial sensors and drone video analysis. Data were collected from 11 elite sit-ski athletes during runs on a designated slope segment. The inertial sensor system showed static accuracy of 2° and an average deviation of 0.008 m, while drone video analysis had a mean relative error of 1.36% ± 0.94%. Analysis of 33 gate turns revealed a mean skiing distance of 13.61 ± 2.87 m and mean time of 0.88 ± 0.19 s, with significant positive correlation ($\:p$< 0.05) between single-gate skiing time (${\it\text{t}}_{\text{s}}$) and the minimum distance (${\it\text{d}}_{\text{m}\text{i}\text{n}}$) ($\:\rho\:$ = 0.74,$\:p$< 0.01) and lateral distance (${\it\text{d}}_{\text{l}\text{e}\text{v}}$) ($\:\rho\:$ = 0.73,$\:p$ = 0.01). Simulation with the inverted pendulum model yielded a COM trajectory length of 97.93 ± 2.31 m, 1.66 ± 3.61 m shorter than actual values ($\:p$ = 0.16), and a simulated time of 6.36 ± 0.64 s, showing strong consistency (ICC = 0.85 for time, ICC = 0.45 for trajectory length). These results confirm that optimizing the balance of turning radius, speed, and distance reduces skiing time, supporting the model’s effectiveness in individualizing trajectory optimization for sit-ski alpine skiing.

## Introduction

Alpine skiing, as a winter sport requiring both technical precision and high speed, has long been a focus of both academic research and performance-oriented training programs. For example, advances in ski-sensor technology and biomechanical analysis have directly improved performance by enabling athletes to fine-tune edge angles and turn dynamics, leading to measurable reductions in race times^1^. Within this context, the design of optimal skiing trajectories not only influences competition outcomes but also serves as a critical component of technical refinement for athletes. A practical example is the carving technique, in which maintaining a clean, continuous edge through a turn depends heavily on selecting a trajectory that balances turning radius, edge angle, and speed control^2,3^.

In recent years, the study of trajectories during gate-turning stages has drawn increasing attention because of their significant impact on skiing speed, distance efficiency, and energy consumption^4^. Research has shown that the rationality of skiing trajectories directly affects an athlete’s performance. For example, although the shortest straight-line path between gates may appear advantageous, factors such as turning radius, speed loss, and energy expenditure often prevent the shortest distance from producing the fastest time^5^. Therefore, the challenge is not simply to minimize distance but to find a trajectory that simultaneously balances speed, distance, and energy expenditure, a core objective of holistic performance optimization^6^.

Various methods for trajectory optimization have been proposed in the existing literature. For instance, Hirano^7^ calculated the fastest skiing path along the fall line by using regression models, while Waegli et al.^8^ conducted dynamic analyses to explore the theoretical foundations of minimum-time trajectories. Pengcheng et al.^9^ and Cai et al.^10^ applied the pseudospectral method to solve optimal skiing trajectories under boundary constraints, and Rudakov et al.^11^ used Newtonian dynamics to optimize slalom trajectories. These studies have validated the theoretical feasibility of trajectory optimization and provided guidance for practice. However, most focus on standing skiing, leaving a significant research gap in the optimization of sit-ski performance.

As a Winter Paralympic event, sit-skiing presents unique challenges due to athletes’ physical constraints and the specialized nature of their equipment. Specifically, effective trajectory planning for carving turns must consider the integrated dynamics of the athlete and the sit-ski apparatus — including unique factors rarely addressed in standing-ski models, such as chassis flex under load, changes in ski–snow contact geometry, and the distinct shift patterns of a seated athlete’s COM during turns^12^. Existing mathematical and kinematic models have not fully captured this coupled dynamic system, leaving open the question of how to optimize COM trajectories for both efficiency and stability in sit-skiing.

Objective and Innovation of this study: To address these challenges, this research develops a modified inverted pendulum model specifically adapted for sit-ski alpine skiing. Unlike conventional applications in standing skiing, our approach incorporates (1) the altered COM height and distribution in the seated posture, (2) the rigid-flexible coupling between athlete and sit-ski equipment, and (3) equipment-specific constraints on edge angle and turning radius. The model is integrated with an inertial sensor and drone-based measurement system to capture high-resolution motion data in real time. Through dynamic modeling and simulation, we aim to analyze gate-turning trajectories, identify key factors influencing performance, and validate the model against actual field performance.​.

Based on the principles of energy conservation in pendulum systems and prior observations of elite sit-skiers, we postulate the following hypotheses:

1. The modified inverted pendulum model will accurately simulate the COM trajectory during gate-turning phases, with a mean relative error of less than 1.5% for distance and no statistically significant difference in time compared with actual measurements.
2. Optimizing the interplay between turning radius, skiing speed, and lateral COM distance will produce a statistically significant reduction in skiing time across gates.
3. Athletes achieving shorter minimum lateral COM distances during turns will demonstrate faster gate-crossing times compared to those with larger deviations.

## Materials and methods

### Study design and participants

This study involved 11 elite sit-ski alpine skiing athletes, all of whom were members of the Chinese national team who participated in the Beijing Winter Paralympics. The participants’ anthropometric characteristics, including height, weight, COM location, classification level, and physical impairment type, are detailed in Table 1.Informed consent was obtained from all participants, and the study was approved by the Jimei University Sports Academy Ethics Committee in accordance with ethical standards for human studies.

**Table 1:** Anthropometric characteristics and classification of sit-ski alpine skiing participants.

Participant	Gender	Height (cm)	Weight (kg)	COM (%)	Classification	Impairment type
LST	Female	165.43	53.44	62.58	LW12	AKr
ZHY	Female	165.54	60.18	60.35	LW11	AKl
ZWJ	Female	160.45	50.43	61.45	LW12	AKl
HSS	Female	160.32	51.12	65.66	LW11	AKr
CHL	Male	175.12	66.33	63.54	LW12	AKl
LZL	Male	167.63	54.2	63.89	LW12	AKl
YHL	Male	171.54	62.25	62.76	LW12	AKr
WAH	Male	165.38	55.45	67.23	LW12	BKr
LIX	Male	170.18	49.25	53.88	LW10	AKl
LLJ	Male	170.58	55.94	54.64	LW11	AKr
GZL	Male	174.41	65.32	59.73	LW10	AKr

Note: LW10–LW12 are IPC Para Alpine Skiing sitting classifications: LW10, markedly limited trunk control/poor seated balance; LW11, partial trunk function/limited seated balance; LW12, normal or near-normal trunk control with primarily lower-limb impairment. AK = transfemoral amputation; BK = transtibial amputation; r/l indicates side. Classifications were assigned by certified classifiers per IPC procedures.

### Experimental environment

Field experiments were conducted on an advanced ski slope at Taiwu Ski Resort, Chongli County, Hebei Province, China. The slope features an altitude of 1,864 m, a total length of 510 m, a vertical drop of 280 m, and an average gradient of 20.8°. The experiments focused on a specific segment of the slope (gates 18–21), characterized by a transverse width of 41.4 m, a slope length of 69.7 m, and a vertical drop of 28.6 m with a 24° incline.

This section was selected because it contains consecutive gates with a relatively consistent gradient and representative turning characteristics, while minimizing environmental variability such as terrain irregularities or snow texture changes, thereby ensuring that the collected kinematic and dynamic data reflect athlete performance under stable and repeatable conditions.

### Testing system and data collection

To address the limitations of conventional single-modality approaches, we developed a drone–inertial sensor integrated kinematic testing system designed for alpine sit-skiing. Existing fixed-camera video methods provide only localized measurements and require complex 3D calibration, while standalone inertial measurement units (IMUs) cannot capture absolute velocities or full trajectory data59,62. Our integrated system combines aerial video coverage with wearable sensors, enabling large-section motion capture and synchronized acquisition of both in-plane trajectory and absolute velocity without the need for full-slope 3D calibration.

The system architecture, mounting strategies, calibration procedures, and error-control measures are detailed in the following subsections.

#### Inertial sensor system

The inertial sensor system was installed on the sit-ski equipment at carefully selected locations to ensure accurate motion tracking under on-slope conditions. The gyroscope/accelerometer module was positioned on the athlete’s dorsal side at the T7 vertebra level—an anatomically stable landmark in seated skiing—using an adjustable elastic harness and EVA foam interface to maintain firm contact and reduce vibration. The differential GPS (dGPS) receiver antenna was mounted on the top surface of the sit-ski leg support frame via a carbon-fiber mounting plate and stainless steel clamp, ensuring a fixed spatial relationship to the ski chassis and an unobstructed view of satellites during motion.

This mounting strategy was chosen to balance biomechanical representativeness with mechanical stability. Nevertheless, potential measurement errors could arise from small relative movements between the athlete’s body and the sit-ski frame, as well as from the fixed sensor locations not coinciding precisely with the instantaneous whole-body COM. To mitigate these effects, secure fixation methods, vibration-damping material, and rigid mounts were applied, and IMU–dGPS data were synchronized to compensate for minor positional offsets over time.

#### Drone video collection system

**Fig. 1:**
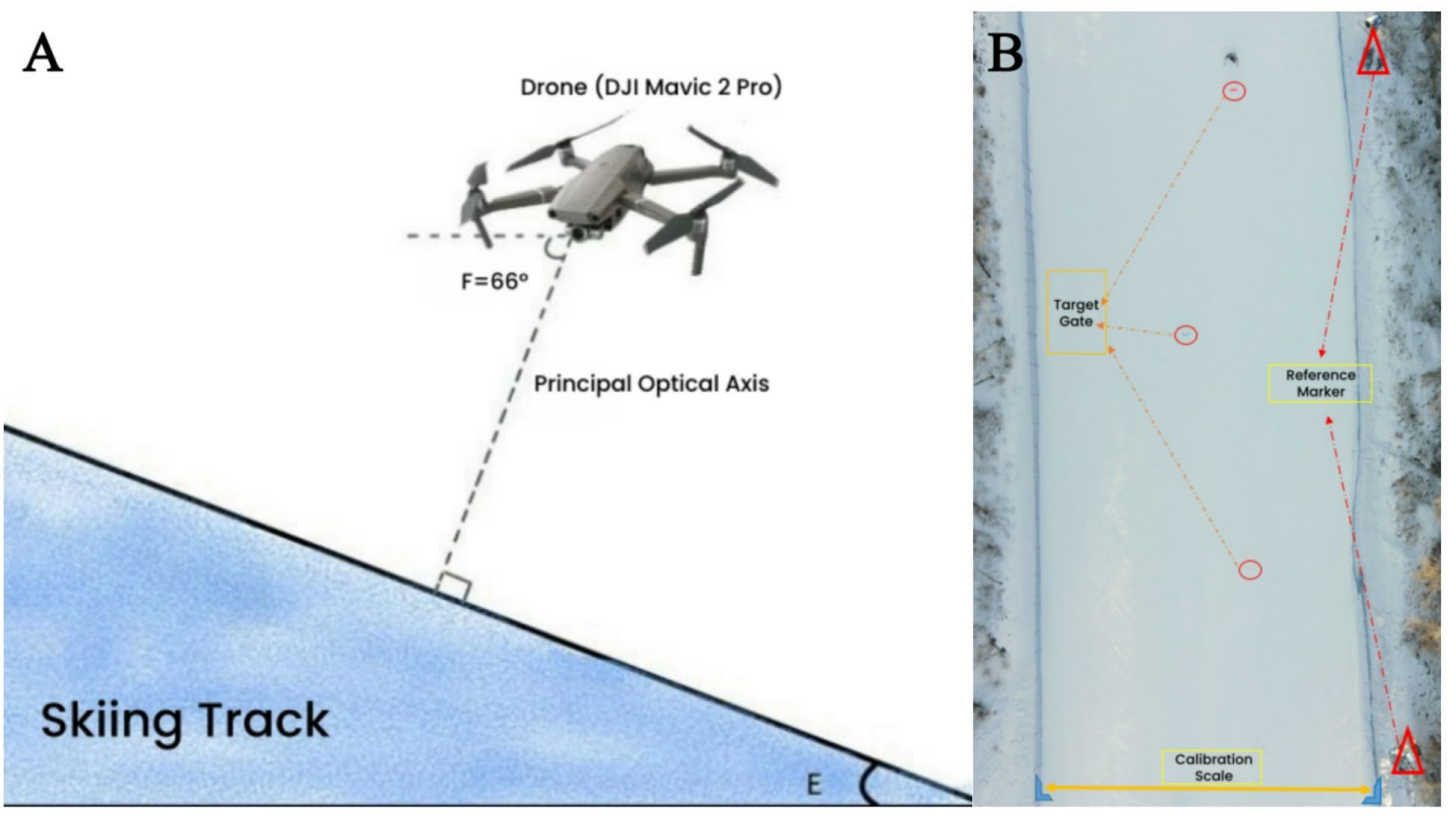
Schematic of the drone-based video collection system. (A) Drone camera setup schematic. (B) Test area calibration schematic.

Video data of the athletes’ skiing performance in the field were collected using a DJI Mavic 2 Pro drone. The camera pitch angle was set to 66° downward (Fig. 1A), ensuring that the optical axis was perpendicular to the target slope. The recording frequency was 60 Hz, providing high‑resolution, multi‑angle footage of the skiing process.

During testing, the drone hovered within a designated flight area over the ski slope to capture stable footage. Data synchronization between drone footage and the IMU–dGPS system followed the method of Brodie et al.^13^. A course‑side signal light was positioned at a designated reference point on the slope. When the athlete passed this point, the light was automatically triggered, producing a distinct visual marker in the UAV video while simultaneously logging the trigger event in the IMU data stream via a photodiode interface. This dual visual–electronic marking enabled precise post‑processing alignment, with laboratory tests showing potential timing errors—arising from trigger detection latency or frame discretization—were < 0.5 frames at 60 Hz (< 8 ms), negligible for the temporal resolution required in this study.

In addition to timing errors, inertial sensors are subject to typical measurement errors^14^, including: (1) offset error—non‑zero output when stationary, which accumulates after double integration for displacement estimation; (2) scale‑factor error—a proportional mismatch between measured and true motion signals; and (3) background white noise—random noise that can cause trajectory drift if uncorrected. To mitigate these, the IMU was firmly secured using a high‑tensile elastic harness, raw data were low‑pass filtered, and UAV video plus optical triggers were used as external reference points for drift correction^4^.

Spatial calibration of the target slope section (Fig. 1B) was performed using a two‑dimensional (2D) planar calibration method. The calibration scale length was precisely measured using a total station (SOUTH NTS‑332R; distance measurement accuracy: ±2 mm + 2 ppm). Several high‑contrast reference markers were strategically placed along the skiing direction to ensure that the calibration scale and reference points were present in every captured frame and to verify scaling accuracy. This 2D method was chosen because the core kinematic variables—such as skiing time along the slope and COM–gate horizontal distances—are defined within the slope plane, and the selected slope segment was smooth with a uniform gradient.

Validation against dGPS COM positions indicated mean spatial error of ± 0.03 m, confirming adequacy for high‑precision in‑plane trajectory analysis. However, 2D calibration cannot capture perpendicular‑to‑slope motion such as jump heights and is therefore unsuitable for slopes with variable gradients or irregular surfaces, where 3D calibration would be required.

### Biomechanical modeling and center of mass measurement

The Center of Mass (COM), a critical parameter in our model, refers to the point in the body where the total mass can be considered concentrated for purposes of motion analysis^15^. In this study, the COM was determined using the segmental method, based on anatomical measurements and dynamic modeling. Segmental mass and segmental COM location values were obtained from our previously published dataset on 17 seated amputee athletes^16^, in which residual limb mass was measured using a force platform, COM locations were determined via balance board testing, and intact limb parameters were calculated using established regression models matched to participant age and body characteristics. The model outputs were validated against balance‑board measurements in that cohort, ensuring suitability for the present study’s sit‑skiing athletes. However, as this dataset was developed for seated amputee athletes, its applicability to non‑amputee sit‑skiers or to extreme high‑dynamics skiing conditions may require further verification.

In sit-skiing, the seated posture results in a lower and more rearward COM compared with standing skiing, influencing both stability and ski–snow interaction^17,18^. To adapt the inverted pendulum model from standing skiing to sit-skiing, three key modifications were introduced:

1. Adjusted COM height and distribution to reflect seated anthropometry;
2. Inclusion of the composite mass and geometry of the sit-ski equipment;
3. Consideration of equipment‑specific constraints such as limited edge angle and turning radius.

The COM trajectory during skiing was monitored in real time using a customized wearable inertial measurement system (WT901WIFI-C model; WitMotion Co., Shenzhen, China) modified for this study’s needs. The IMU modules comprised a tri‑axial accelerometer, a tri‑axial gyroscope, and a tri‑axial magnetometer. The accelerometer measured linear accelerations along three orthogonal axes, the gyroscope recorded angular velocities, and the magnetometer provided orientation reference relative to the Earth’s magnetic field. The IMU module was mounted near the mid‑back, close to the estimated COM, and aligned with the body’s longitudinal axis using a high‑tensile elastic harness to minimize relative movement; the differential GPS receiver was affixed to the sit‑ski frame using hook‑and‑loop fasteners.

Calibration of the IMU system was conducted using a rotary table (WMD3‑K) with an AS5048A angular sensor (resolution 0.0219°, accuracy 0.05°). IMU and reference angles were read simultaneously via the same microcontroller at 100 Hz, with a time-sync error < 10 ms. Static and dynamic step‑rotation tests across the X, Y, and Z axes were used to quantify measurement accuracy before field deployment. To further reduce noise and drift, raw signals were low‑pass filtered, and the secure mounting helped minimize soft‑tissue motion effects.

The system output skier posture in both Euler angles and quaternions, enabling synchronized comparison between sensor-derived COM position/orientation and the COM trajectory predicted by the model.

### Simulation of skiing trajectories based on the inverted pendulum model

In alpine skiing, skiing trajectories consist of both the ski path and the motion trajectory of the body’s COM. In practice, some athletes focus excessively on maximizing skiing speed while neglecting another critical factor that affects skiing time—trajectory length. A well‑designed trajectory not only helps maintain a stable skiing speed but also enables athletes to better preserve balance.

Carving, one of the most widely used techniques for gate turns in alpine skiing, is preferred because it minimizes speed loss during turns and offers strong maneuverability. The inverted pendulum model has been widely applied in skiing dynamics studies^13^, as it captures the essential relationship between body inclination and turn dynamics, making it theoretically feasible for simulating sit‑ski alpine skiing trajectories.

In sit‑skiing, during a carving turn, the athlete leans inward to generate the centripetal force necessary for curved motion. This mechanical characteristic corresponds closely to the motion mechanism of an inverted pendulum: the pendulum’s inclination angle directly reflects the torque and resulting acceleration experienced by the combined skier–equipment COM. In this study, the athlete and the sit‑ski were treated as a unified rigid body, and the gate‑turning phase was simplified to an inverted pendulum model, following the approach of Brodie et al.^13^. The model’s deflection angle (body lean angle) was set as the key control variable for steering.

Using Newton’s second law, we formulated the dynamic equations of the system under the assumption of carving turns. Boundary constraints were defined according to course and athlete parameters, and the trajectory optimization problem was transformed into an optimal control problem with the objective of minimizing skiing time. This modeling approach was considered valid for the present context because it:

1. directly links the control variable (lean angle) to actual skier posture, enhancing the practical relevance of the optimization results;
2. accommodates athlete‑specific anthropometric and dynamic parameters while accounting for slope and environmental constraints, improving the reliability and applicability of the simulated trajectories.

#### Development of the mathematical model

Carving turns were applied at the three selected gates based on skiing footage to ensure that the simulation results closely aligned with actual skiing performance. In these turns, no relative sliding or aerial phases were observed. The system comprising the athlete and the sit‑ski was selected as the object of study. The mathematical model (Eqs. 1 and 2) was developed under the following key assumptions: (1) the skis were regarded as rigid bodies with no elastic deformation; (2) within each individual turn, the system was in a quasi‑steady‑state speed condition, neglecting transient acceleration and deceleration phases; and (3) the slope surface was homogeneous hard‑packed snow with a constant coefficient of sliding friction (*µ*). These simplified assumptions were necessary to reduce the complex nonlinear system into a tractable optimal control problem focused on the influence of body‑lean angle on trajectory and performance. However, they may also limit the generalizability of the results: neglecting ski deformation may overlook performance effects in high‑level racing turns that utilise ski flex; the steady‑state speed assumption omits non‑steady dynamics in tight sequences of turns; and the constant‑friction assumption does not represent changes in snow conditions over time.

**Fig. 2:**
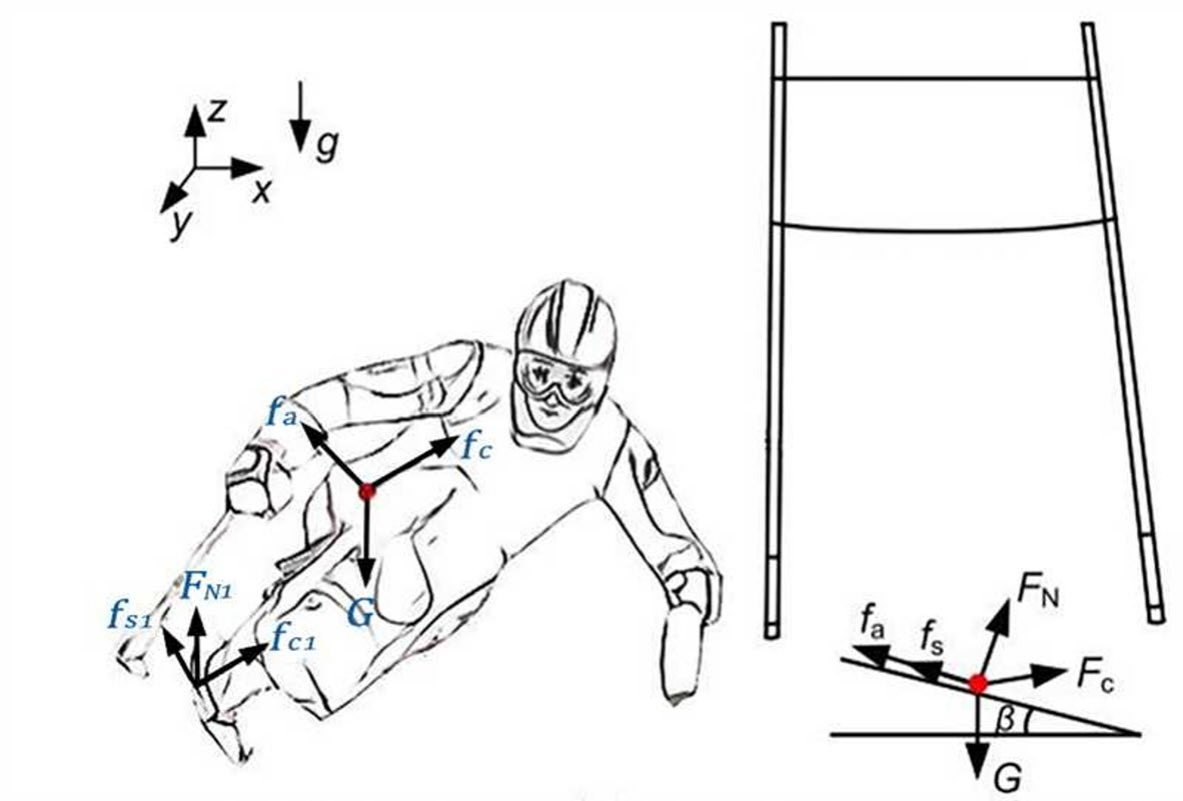
Force analysis during gate turning.

Within the skiing trajectory coordinate system, kinematic equations were established on the basis of Newton’s second law and the formula for centripetal force (Eqs. 1 and 2). The main variables and their determination methods are as follows:

1. *G*: Calculated from total system mass (athlete + equipment, measured directly) and gravitational acceleration $g=9.8m/{s^2}{\text{~}}\left( {G=m \cdot g} \right)$.
2. ${\beta _1}$: Obtained from official course topographic data and verified with field surveying (total station), measured as 24° for the study course.
3. ${\rho _a}$: Computed from site altitude (1800 m) and average competition‑period temperature (–10 °C) via the ideal gas law, giving ~ 1.0525 kg/m^3^.
4. $\mu$: Taken from literature values for alpine skiing on hard‑packed snow^19^, set to 0.20.
5. ${C_d}$ and *s*: Estimated based on wind‑tunnel data for winter sports aerodynamics^20^, using seated skier posture combined with sit‑ski geometry to determine ${\it{\text{C}}_{\text{d}}} \cdot {\it\text{s}}$.
6. ${F_c}$: Calculated within the model from mass, velocity, and radius of curvature (${{\it\text{F}}_{\it\text{c}}}={\it\text{m}}{{\it\text{v}}^2}/{{\it\text{R}}_{\it\text{c}}}$), not an independent input.
7. ${R_c}$: Obtained from the optimized trajectory geometry determined by the control variable ${\varphi _1}$.
8. ${\varphi _1}$: Measured in the field using a wearable inertial measurement unit (IMU) mounted near the athlete’s COM, with tri‑axial accelerometer, gyroscope, and magnetometer signals fused to estimate tilt angle in real-time.

1 $$\left\{ {\begin{array}{*{20}{c}} {m{{\ddot {x}}_1}= - {F_c}\;{\text{sin}}{\varphi _1} - \left( {{f_a}+{f_s}} \right)\;{\text{cos}}{\varphi _1}} \\ {m{{\ddot {y}}_1}=G^{\prime}+{F_c} - \left( {{f_a}+{f_s}} \right)\;{\text{sin}}{\varphi _1}} \\ {G^{\prime}=mg\;{\text{sin}}{\beta _1}} \\ {{f_a}=0.5{C_d}{\rho _a}s{v^2}} \\ {{f_s}=\mu mg\;{\text{cos}}{\beta _1}} \end{array}} \right.$$

2 $$\left\{ {\begin{array}{*{20}{c}} {m\dot {v}=G^{\prime}\;{\text{sin}}\varphi - \left( {{f_a}+{f_s}} \right)} \\ {{F_c}+G^{\prime}\;{\text{cos}}\varphi \;{\text{sin}}\dot {\varphi }=m\frac{{{v^2}}}{{{R_c}}}} \end{array}} \right.$$

where *G* represents the gravitational force, $G'$ represents the component of *G* acting perpendicular to the slope surface, ${f_a}$ represents the air resistance, ${f_s}$ denotes the sliding friction force between the skis and the snow surface, $\mu$ indicates the coefficient of friction, ${F_c}$ refers to the centripetal force, ${C_d}$ represents the drag coefficient, ${\rho _a}$ denotes the air density, *s* indicates the frontal area, ${R_c}$ refers to the turning radius, ${\varphi _1}$ represents the athlete’s body inclination angle, and ${\beta _1}$ denotes the slope angle of the course. The force analysis during the gate-turning process is illustrated in Fig. 2.

#### Definition of the ideal turning model and boundary constraints

**Fig. 3:**
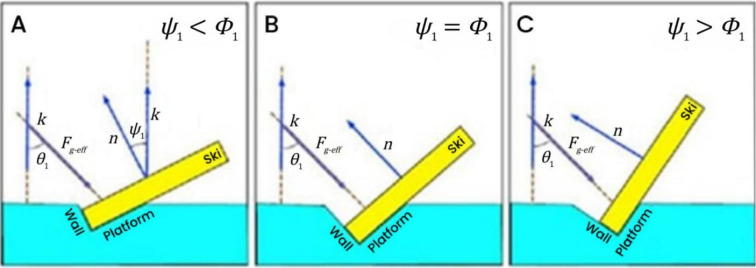
Schematic of the relationship between ski edge angle and gate-turning dynamics.

The combined force of the gravitational component and centripetal force was defined as the equivalent gravitational force ${F_{g - eff}}$. From the perspective of force balance analysis, the following scenarios were considered:

1.When the edge angle of the ski is considerably small, the friction force between the ski and the snow surface is less than the centripetal force, i.e., ${{\it\Psi} _1}<{{\it\Phi} _1}$ (Fig. 3A). In this case, the ski will slide sideways, making it impossible to execute the turn using carving techniques.
2.When the edge angle of the ski is excessively large, the friction force between the ski and the snow surface exceeds the centripetal force, i.e., ${{\it\Psi} _1}>{{\it\Phi} _1}$ (Fig. 3C). Although the turn can be executed using carving techniques, the excess friction will cause a loss of speed.
3.An ideal turn occurs when the friction force equals the centripetal force, i.e., ${{\it\Psi} _1}={{\it\Phi} _1}$ (Fig. 3B). In this scenario, the turn can be executed using carving techniques without any loss of speed, achieving optimal performance.

**Fig. 4:**
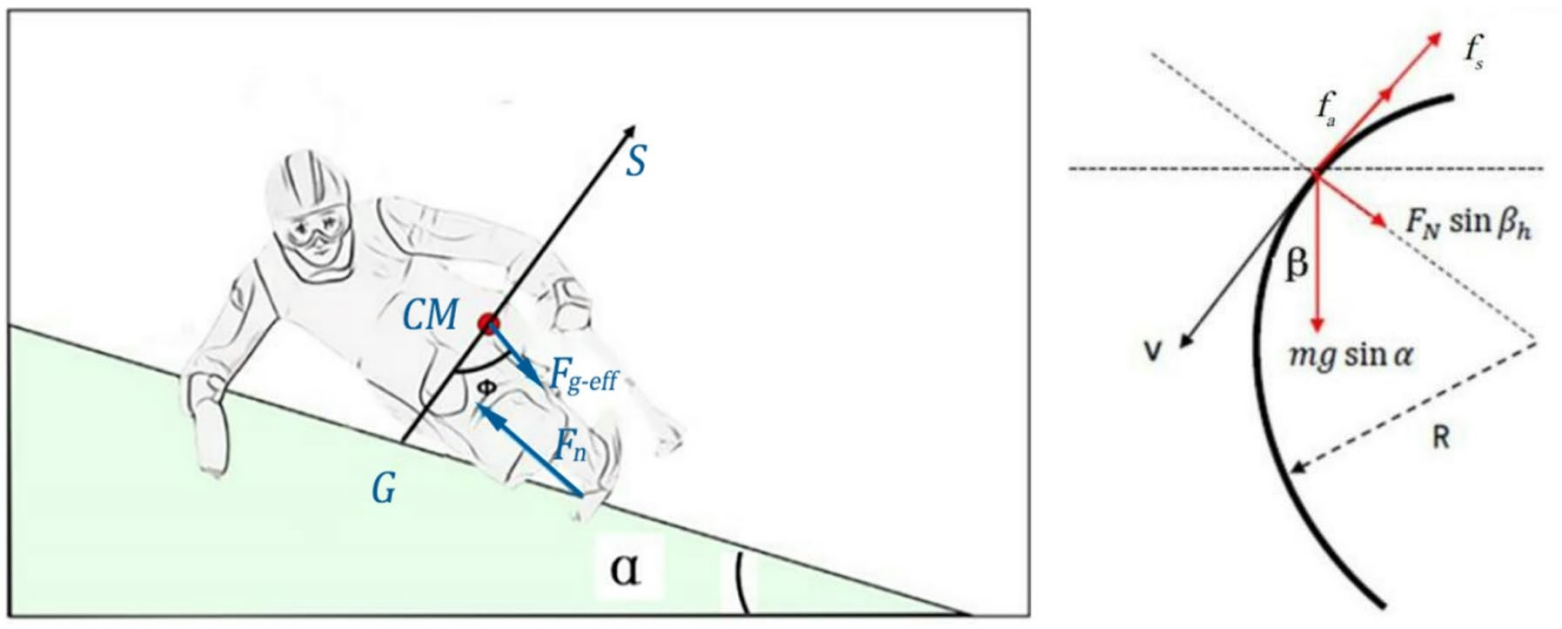
Force diagram of the carving turn system.

Assuming the athlete and the sit-ski form a rigid body, with the ground reaction force acting through the inner edge of the ski and passing through the COM, and the turn being a carving turn without lateral slipping (Fig. 4), the constraint equation for the ideal turn is

3 $${\text{tan}}{{\it\Phi} _1}=\frac{{{v^2}}}{{g{R_c}}}\frac{1}{{{\text{cos}}\alpha }} - {\text{tan}}\alpha {\text{cos}}\beta$$

where ${{\it\Phi} _1}$ denotes the angle between the line connecting the COM to the inner ski edge and the normal (vertical) to the slope surface; $\alpha$ is the slope angle of the course, defined as the angle between the slope surface and the horizontal ground; and $\beta$ is the body inclination angle, defined as the angle between the athlete’s trunk axis (passing through the COM and pelvis) and the normal to the slope; and *v* represents the skiing speed. Geometrically, these three angles are interrelated through the slope geometry and body posture (see Fig. 4).

For any segment of the COM trajectory, based on Newton’s second law, the following equations can be derived in the skiing trajectory coordinate system:

4 $$\left\{ {\begin{array}{*{20}{c}} {mgsin\beta cos\alpha - {f_a} - {f_s}=m{a_\tau }} \\ {{F_N}cos\alpha =mgcos\beta } \end{array}} \right.$$

where ${F_N}$ represents the normal force exerted by the snow surface on the ski, ${a_\tau }$ represents the centripetal acceleration, ${f_a}$ represents the air resistance, and ${f_s}$ denotes the sliding friction force between the skis and the snow surface. The constraint condition is that ${F_N}$ must not exceed the lateral force exerted by the snow surface on the ski; otherwise, sideway sliding will occur during the turn, resulting in a loss of speed or failure to complete the turn using carving techniques.

For capturing a more complete gate-turning trajectory, the trajectory optimization phase was defined as the period from the zero-crossing point of the athlete’s body inclination angle before entering the first target gate to the zero-crossing point after passing the last target gate. Through inputting the athlete’s basic parameters and course parameters, the models were constructed using Functions 1, 2, and 4, with the constraint condition established by Eq. (3). This approach allowed the simulation of trajectories and calculation of key target variables, such as skiing distance and time.

### Data processing and analysis

The drone-captured video data were analyzed using KX Motion video analysis software (Version 1.0; Beijing Senmiaoxin Sports Technology Co., Ltd., China). Note: The software provider has since ceased operations, and therefore an active URL is no longer available. This software was used to perform two-dimensional decomposition, extracting key parameters such as the COM trajectory, skiing time, and velocity. These data were synchronized and matched with the GPS data recorded by the inertial sensor system. The COM trajectory and skiing path for the target section were determined using the coordinates of the gates. The curvature radius (R) of the COM trajectory was calculated in MATLAB 2014 using a three-point sliding window method. This approach fits second-order polynomials locally to the x- and y-coordinates within each window as functions of arc length. The curvature (κ) was then computed analytically from the derivatives of these fitted polynomials using the standard parametric form, and the radius was derived as *R* = 1/|κ|. This local fitting approach effectively smoothed the trajectory and mitigated the impact of discrete data noise on curvature estimation. The resulting curvature features were analyzed to assess the effect of turning radius on skiing performance.​.

For statistical analysis, IBM SPSS Statistics (Version 21.0; https://www.ibm.com/products/spss-statistics) was used to perform a comprehensive examination of the data. The accuracy of the drone-based measurements across the 11 trials was first compared using a one-way repeated-measures ANOVA, treating each trial as a repeated measurement under identical experimental conditions. Subsequently, correlations between skiing time, COM trajectory features, and skiing performance metrics. Prior to applying Pearson correlation analysis to continuous variables (e.g., skiing time, skiing distance, turning radius), the assumptions of normality and homoscedasticity were assessed. Normality was verified using the Shapiro–Wilk test (*p* > 0.05 for all variables), and homoscedasticity was evaluated using the White test, confirming that all continuous variables met these assumptions. Therefore, Pearson correlation analysis was applied to continuous variables, whereas Spearman correlation analysis was used for relationships between categorical variables (e.g., technical classifications) and skiing performance.

To validate the accuracy of drone‑captured data, analytical results (e.g., COM trajectory length, skiing time) were compared against “actual measurements” obtained from a differential GPS (dGPS) system, which provides high‑accuracy COM positions and timing data under field conditions. The dGPS system operates with a differential correction method to ensure centimeter-level accuracy in position measurements. The actual measurements included the COM position and timing data, collected in real-time as the participants skied the course. These values served as the reference data for validating the drone-derived measurements.

Single‑sample *t*‑tests were performed to evaluate the agreement between the drone-derived measurements (e.g., COM trajectory length, skiing time) and the dGPS reference data. Specifically, the distance obtained from drone video analysis was compared with the actual distance measured by the dGPS system to assess the reliability of the drone-based measurements.

For model validation, paired‑sample *t*‑tests were used to compare the COM trajectory length and skiing time computed from the inverted pendulum model with the actual values measured by dGPS and on‑course timing gates, to assess the agreement between the simulated and real-world data. The significance level was set at *p* < 0.05, and the highly significant level at *p* < 0.01.

## Results

### System testing results and accuracy analysis

**Fig. 5:**
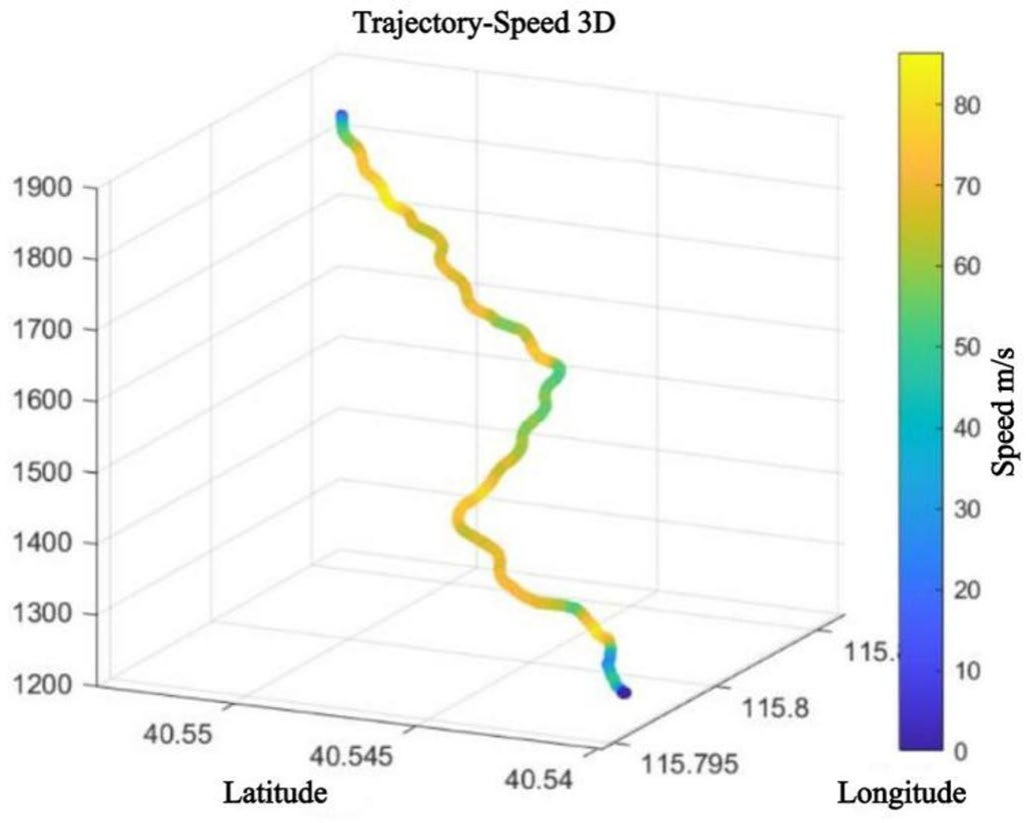
Full trajectory of a sit-ski giant slalom run.

The kinematic testing system developed in this study demonstrated high precision and reliability during actual skiing trials. Through the integrated application of inertial sensors and the drone system, the skiing trajectories and relevant kinematic indicators of athletes were successfully recorded. Figure 5 illustrates the full COM trajectory of a sit-ski giant slalom run in the target section.

In terms of system accuracy validation, the results from turntable experiments indicated that the inertial sensor system had a static accuracy of 2°, a drift value of 6.3 cm, and a magnetic declination of 3.18°. The average deviation between the static and real-time kinematic modes was 0.008 m, with a maximum deviation of 0.029 m. Based on a three-dimensional calibrated video analysis under similar skiing conditions, the maximum difference between the gyro-measured lateral inclination angle and the video analytical result was 3.5°.

Table 2 presents the accuracy validation results of the drone video analysis. In addition to calculating the mean relative error (1.36% ± 0.94%) and conducting a single‑sample t‑test against dGPS‑measured reference values, we examined the error values across the 11 trials for potential outliers and systematic variation. Outlier screening using both box‑plot analysis and z‑score thresholds (|z|>3) identified no extreme values; Shapiro–Wilk tests confirmed that error values were normally distributed (*p* > 0.05), and a one‑way repeated‑measures ANOVA found no significant variation in accuracy between trials (*p* = 0.47). These results indicate that the drone‑based system provided consistently high measurement reliability across all tested runs.

**Table 2:** Accuracy statistics of drone video analysis.

Serial number	Measured distance (m)	Analyzed distance (m)	Error (m)	Relative error (%)
1	71.5	70.41	1.09	1.52
2	71.5	70.28	1.22	1.71
3	71.5	71.08	0.42	0.59
4	71.5	73.86	2.36	3.3
5	71.5	73.61	2.11	2.95
6	71.5	72.3	0.8	1.12
7	71.5	71.76	0.26	0.36
8	71.5	72.39	0.89	1.25
9	71.5	71.98	0.48	0.67
10	71.5	72.35	0.85	1.19
11	71.5	71.75	0.25	0.35
Mean ± SD	71.50 ± 0.00	71.98 ± 1.08	0.98 ± 0.67	1.36 ± 0.94

The results indicated that the testing system developed in this study could meet the data collection requirements for high‑precision, synchronised kinematic and positional measurements of the skier’s COM under complex on‑slope conditions.

### Characteristics of skiing time and distance for single gate turns

The COM trajectory represents the actual skiing path chosen by the athlete based on the gate positions, terrain features, and their technical skill level. This study analyzed the skiing time, skiing distance, and the spatial relationship of the COM trajectory for each gate in the target section. The descriptive statistics, including means, standard deviations, and 95% confidence intervals, are presented in Table 3.

**Table 3:** Statistics of skiing distance and time for single gate turns.

	Min.	Max.	Mean	SD	95% CI for Mean	
					Lower	Upper
${{{\it\text{L}}}_{\text{s}}}$(m)	7.97	19.41	13.61	2.87	12.56	14.66
${\it\text{t}}_{\text{s}}$(s)	0.58	1.25	0.88	0.19	0.81	0.95
${\it\text{d}}_{\text{m}\text{i}\text{n}}$(m)	0.49	2.52	1.31	0.47	1.14	1.48
${\it\text{d}}_{\text{l}\text{e}\text{v}}$(m)	0.49	2.68	1.34	0.49	1.16	1.52

Note: ${{{\it\text{L}}}_{\text{s}}}$ is the skiing distance for a single gate, ${\it\text{t}}_{\text{s}}$ is the skiing time for a single gate, ${\it\text{d}}_{\text{m}\text{i}\text{n}}$ is the minimum distance between the COM and the gate, and ${\it\text{d}}_{\text{l}\text{e}\text{v}}$ is the lateral distance at the moment of reaching the gate. The sample size is *n* = 33.

As shown in Table 3, the mean skiing distance for a single gate was 13.61 ± 2.87 m (95% CI [12.56, 14.66]), and the mean skiing time was 0.88 ± 0.19 s (95% CI [0.81, 0.95]). The confidence intervals indicate the precision of these mean estimates. Slight variations in skiing distance and time were observed between different gates, and these variations were closely related to the spatial positioning of the gates. This observation underscores the influence of course design on athlete performance and provides a rationale for further investigating the relationship between trajectory choice and skiing efficiency, as explored in the following section on correlation analysis.

**Fig. 6:**
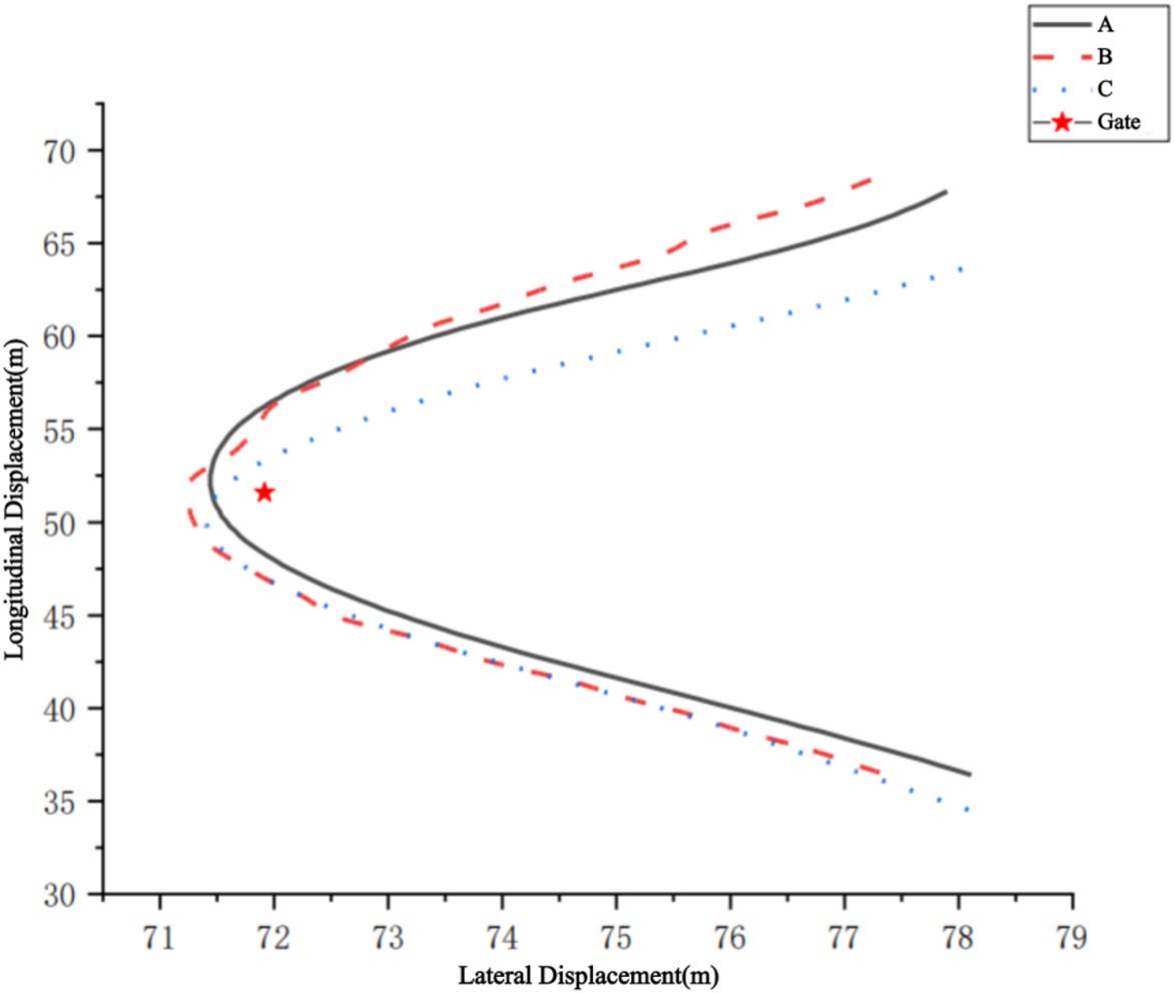
Three types of COM trajectories during gate turning.

Further analysis revealed that the minimum distance between the COM and the gate (${\it\text{d}}_{\text{m}\text{i}\text{n}}$) and the lateral distance of the COM relative to the gate (${\it\text{d}}_{\text{l}\text{e}\text{v}}$) effectively reflect the rationality of the chosen skiing trajectory. The spatial relationship between the COM and the gate exhibited three main trajectory patterns:

1. Turning completed before reaching the gate (Fig. 6A): the minimum distance occurred after passing the gate.
2. Turning completed at the gate position (Fig. 6B): the minimum distance occurred at the gate.
3. Turning completed after passing the gate (Fig. 6C): the minimum distance occurred before reaching the gate.

It is noteworthy that these three COM trajectory patterns differ from those typically reported in standing‑ski literature. In standing alpine skiing, particularly in technical disciplines, turns are more often completed before or exactly at the gate, leveraging active lower‑limb edging and flexion–extension to manage line choice. In contrast, our sit‑ski athletes more frequently exhibited the “after‑gate” completion pattern, likely due to constraints such as reduced lower‑limb control, reliance on upper‑body inclination, and the mechanical characteristics of the sit‑ski frame. This tendency suggests that optimal sit‑ski techniques may prioritize maintaining stability through the gate line before completing the turn, in contrast to the earlier completion strategies often favoured in standing skiing.

### Correlation analysis between skiing time and trajectory characteristics

**Fig. 7:**
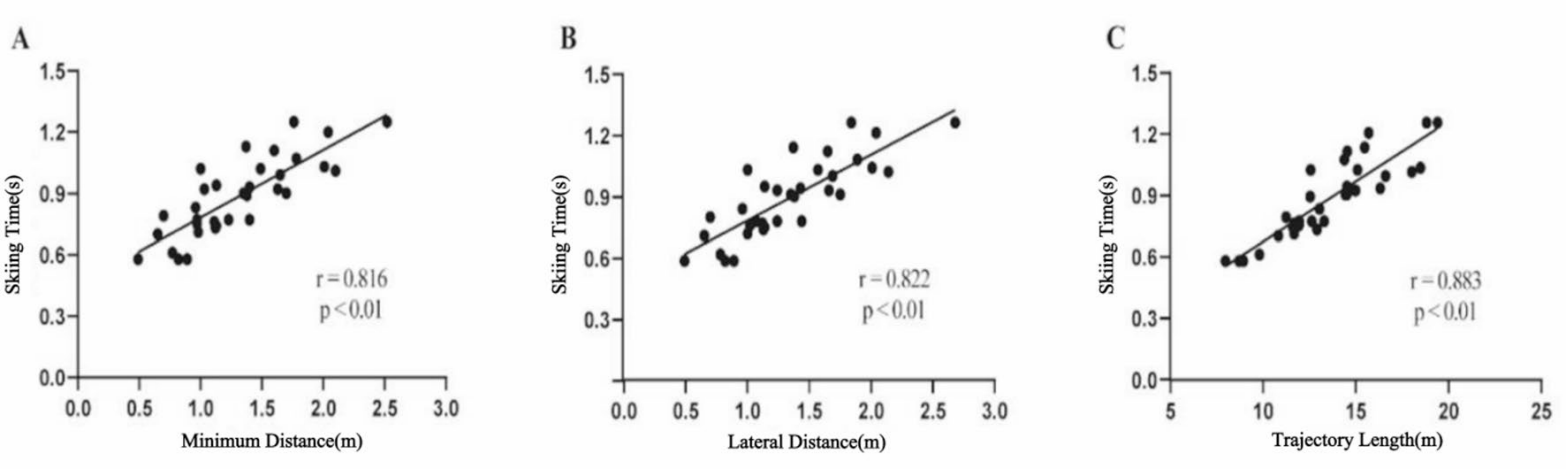
Correlation analysis between single-gate skiing metrics and skiing time. The skiing time in this figure refers to the single-gate skiing time (${\it\text{t}}_{\text{s}}$).

To further investigate the effect of skiing trajectory on skiing time, this study analyzed the correlations between single-gate skiing time (${\it\text{t}}_{\text{s}}$), the minimum distance between the COM and the gate (${\it\text{d}}_{\text{m}\text{i}\text{n}}$), the lateral distance (${\it\text{d}}_{\text{l}\text{e}\text{v}}$), and the skiing distance (${{{\it\text{L}}}_{\text{s}}}$). These relationships are visualized in Fig. 7, which presents scatter plots where each data point (*n* = 33) represents one gate turn, with a linear regression line and the Pearson correlation coefficient (*r*) and p-value displayed for each pair of variables. The analysis revealed strong positive correlations (all *p* < 0.01) between single-gate skiing time and all assessed trajectory characteristics. Specifically, ${\it\text{t}}_{\text{s}}$ showed the strongest correlation with skiing distance (${{{\it\text{L}}}_{\text{s}}}$, *r* = 0.883), followed by lateral distance (${\it\text{d}}_{\text{l}\text{e}\text{v}}$, *n* = 0.822) and minimum distance (${\it\text{d}}_{\text{m}\text{i}\text{n}}$, *r* = 0.816).​

The analysis results showed a significant positive correlation (*p* < 0.05) between single-gate skiing time (${\it\text{t}}_{\text{s}}$) and the minimum distance (${\it\text{d}}_{\text{m}\text{i}\text{n}}$) ($\rho$ = 0.74, *p* < 0.01) and lateral distance (${\it\text{d}}_{\text{l}\text{e}\text{v}}$) ($\rho$ = 0.73,*p* = 0.01). That is, shorter minimum distances and shorter lateral distances contributed to reduced skiing time.

**Fig. 8:**
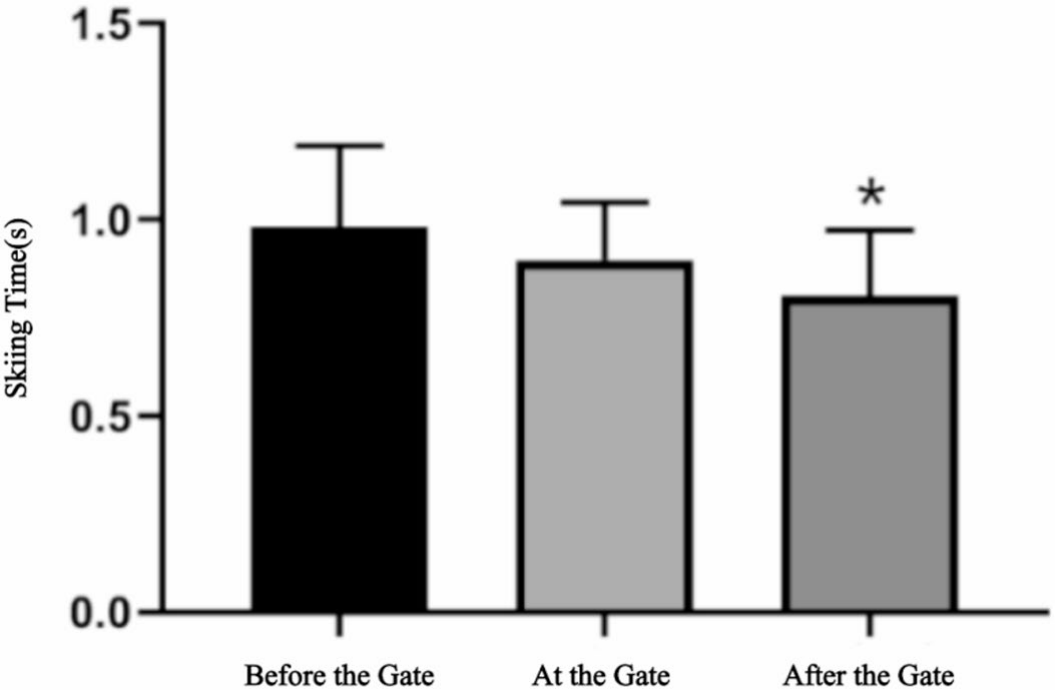
Comparison of skiing time across different gate-turning phases. The skiing time in this figure refers to the single-gate skiing time (${\it\text{t}}_{\text{s}}$).

The Spearman correlation analysis further revealed a significant negative correlation ($\rho$ = −0.42,*p* = 0.02) between skiing time and the position where the minimum distance occurred. Specifically, skiing trajectories where the minimum distance (${\it\text{d}}_{\text{m}\text{i}\text{n}}$) occurred after passing the gate resulted in significantly shorter skiing times compared with those where the minimum distance occurred before or at the gate (Fig. 8).

### Relationship between turning radius and gate-turning performance

**Fig. 9:**
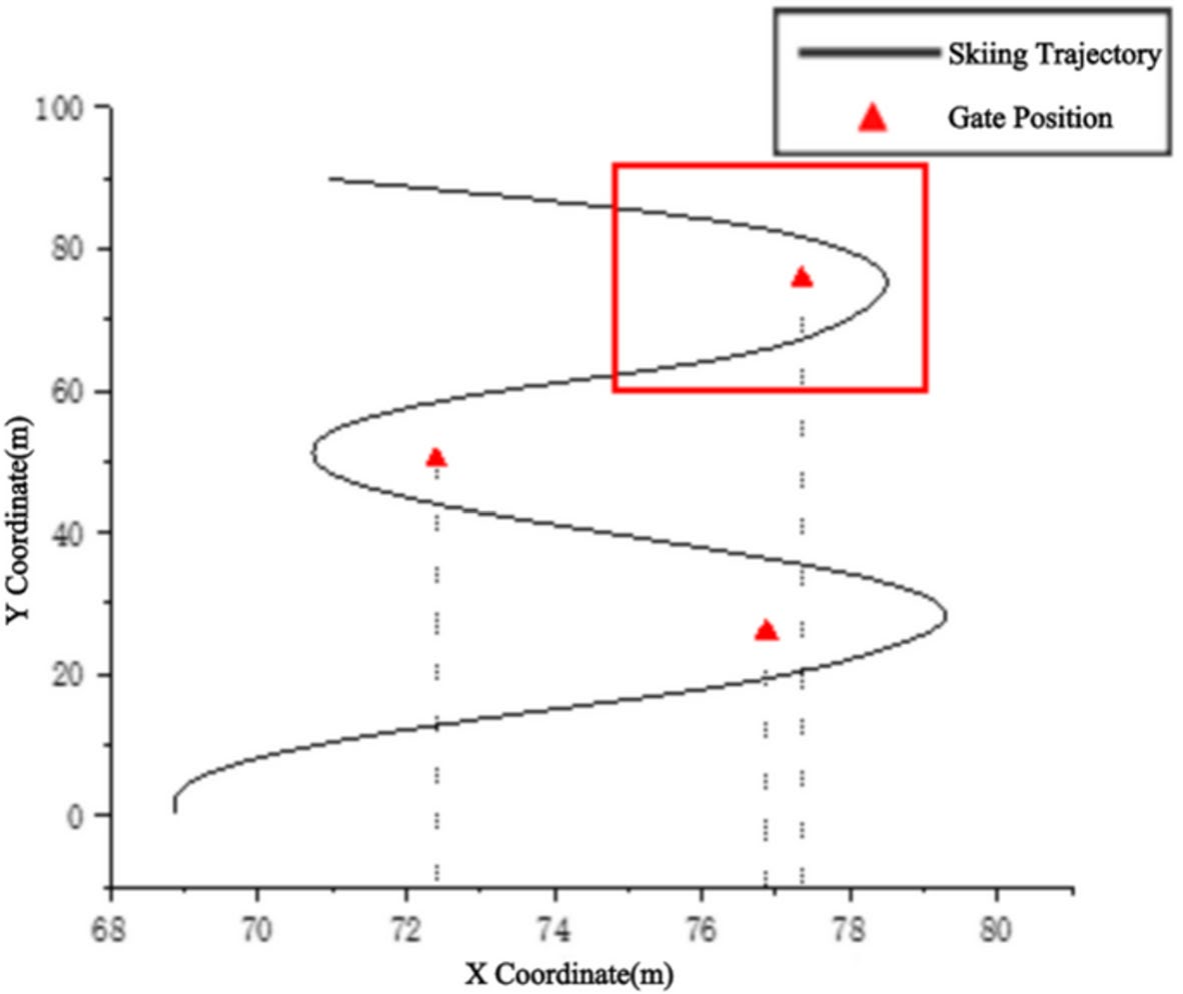
COM trajectory extraction via video analysis and turning radius calculation.

On the basis of the calculation method for turning radius, the COM trajectories within the target section were obtained through video analysis of the athletes (Fig. 9). The trajectory curvature was determined by fitting the trajectories with a quadratic polynomial.

### Simulation results of COM trajectory during gate-turning phases

For achieving the research objectives, the body posture parameters and skiing data collected in the field were integrated with the environmental parameters of the slope and input into the inverted pendulum model to simulate the COM trajectory and skiing time. Basic parameters included the athlete’s height and weight, the weight of the sit-ski equipment, the weight of the skis, and the slope angle of the course. Air density ($\rho$ = 1.0525 kg/m^3^) was calculated on the basis of the altitude of approximately 1800 m and an air temperature of − 10 °C, while the coefficient of friction between the snow and skis ($\mu$ = 0.2) was obtained from the literature^19^.

**Fig. 10:**
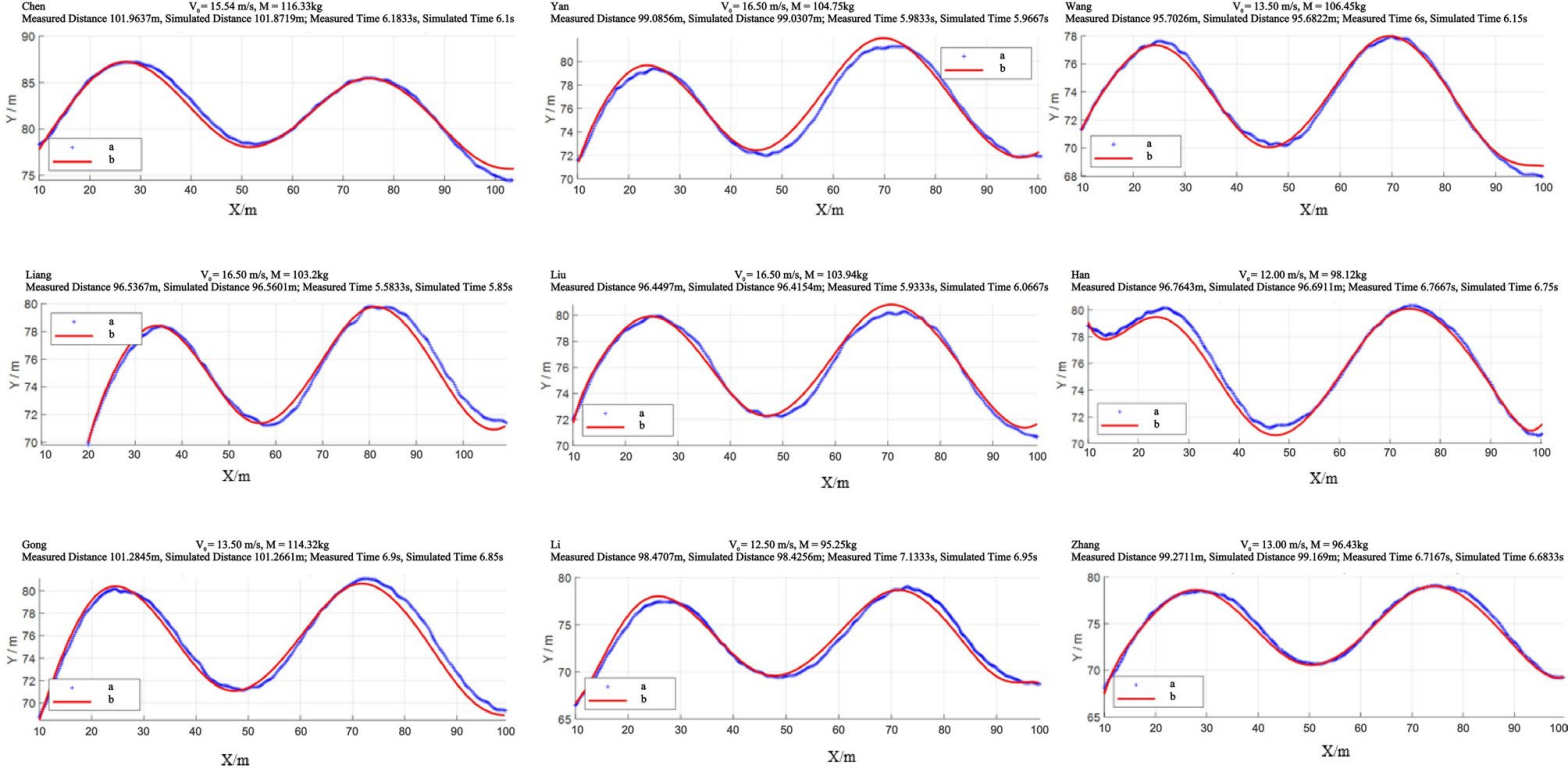
Simulation performance of the COM trajectory model Line a denotes the measured COM trajectory; line b denotes the simulated trajectory. Axes: X, downslope distance along the slope plane (m); Y, lateral offset across the slope plane (m).

For the slope section with a continuous angle of 24°, characteristic parameters were manually set, and simulation accuracy was validated using field-measured data. The computational program was adjusted accordingly. The final simulation results are shown in Fig. 10.

**Fig. 11:**
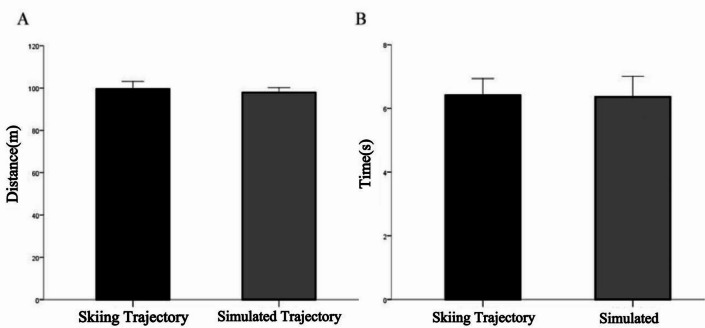
Comparison of simulation results with actual measurements.

From Fig. 11, the length of the COM trajectory calculated using the inverted pendulum model was 97.93 ± 2.31 m, which was 1.66 ± 3.61 m shorter than the actual measured trajectory. A paired t-test revealed no significant difference between the simulated and actual trajectory lengths (*p* = 0.16). Similarly, the simulated skiing time was 6.36 ± 0.64 s, which was 0.06 ± 0.42 s shorter than the actual skiing time, with no significant difference observed (*p* = 0.67).

Additionally, the interclass correlation coefficient (ICC) analysis showed moderate agreement between the actual and simulated trajectory lengths (ICC = 0.45). For skiing time, the simulation results demonstrated excellent consistency with the actual measurements (ICC = 0.85). The lower ICC value for trajectory length likely reflects multiple factors. First, the inverted pendulum model simplifies certain biomechanical aspects (e.g., dynamic edging, ski deformation, and snow interaction), which may reduce its ability to replicate fine-scale variations in path length. Second, although the drone-based measurement system has been validated as reliable, small cumulative errors in distance extraction could influence length calculations more than time estimates. Finally, natural variability in athletes’ steering and upper-body inclination introduces fluctuations in the actual trajectory that the model, which assumes consistent control inputs, cannot fully capture. Taken together, these considerations suggest that while the model reproduces skiing time with high fidelity, further refinement is required to improve its accuracy in estimating trajectory length.

## Discussion

### Role of the inverted pendulum model in sit-ski alpine skiing trajectory simulation

This study validated the effectiveness of the inverted pendulum model in simulating the gate‑turning trajectories of sit‑ski alpine skiing. A comparative analysis between the simulated results for skiing distance and skiing time and the actual measured data revealed no significant differences, and the model demonstrated excellent consistency in skiing time (ICC = 0.85). These findings indicate that the inverted pendulum model effectively captured the characteristics of the athlete’s COM trajectory, thereby providing strong support for our first hypothesis and confirming its suitability for kinematic analysis in sit‑ski alpine skiing.​.

A detailed comparison of the simulation outputs with the actual measurements offers further insight. The lack of a statistically significant difference underscores the model’s utility for predicting overall performance metrics like time and distance. It is noteworthy, however, that the model produced a consistent, albeit non-significant, shortening of the trajectory (by 1.66 ± 3.61 m). This slight underestimation may stem from the model’s simplification of the skier-equipment system as a rigid body, neglecting subtle steering adjustments and ski-snow interaction dynamics that can slightly elongate the real path. Furthermore, the higher consistency for time prediction (ICC = 0.85) compared to trajectory length (ICC = 0.45) suggests that the model excels at capturing the temporal dynamics of the turn—which is crucial for performance—even if the precise spatial path has more variability. This reinforces that the model is particularly well-suited for analyzing key performance factors related to speed and timing.​.

Compared with existing studies, this research introduces innovative advancements in the specific application of sit‑ski alpine skiing. For instance, Rudakov et al.^11^ developed an optimization model for skiing trajectories in traditional standing alpine skiing based on Newton’s second law, while Dźbski^21^ analyzed skier dynamics using principles of classical mechanics. More recently, Komissarov^22^ proposed a theoretical framework for perfect carving turns using a basic centrifugal inverted pendulum model. This model, subject to gravitational and centrifugal forces, describes subcritical and supercritical regimes of skier balance, and was shown—through World Cup race data—to reflect the dynamic skiing behaviours of elite racers, particularly in the supercritical regime.

In the context of biomechanical measurement, Gilgien et al.^23^ demonstrated the use of a lightweight differential GNSS system to quantify external forces in alpine skiing under actual race conditions with high repeatability and precision, enabling meaningful comparisons between skiers and turns. Complementing this, Nemec et al.^24^ applied machine learning methods to high‑precision GNSS data to estimate skier COM and ski trajectories more accurately than the oversimplified inverted‑pendulum body models, using a nine‑degree‑of‑freedom representation of the skier.

Beyond single‑rigid‑body approaches, Zhang et al.^25^ introduced a three‑rigid‑body‑particle model for Super‑G skiing, combining detailed biomechanical representation with Radau pseudospectral optimization to study the effects of body type, joint strength, and turn radius on performance. Similarly, Cai & Yao^10^ formulated a multi‑phase nonlinear optimal control problem for Super‑G trajectory optimization under equality and inequality constraints, solving for time‑minimal racing lines. Although these models provide richer biomechanical detail, they require extensive parameter input and computational resources.

By contrast, the present study extends the inverted pendulum concept into the unique context of sit‑skiing, where the athlete and equipment form a coupled dynamic system. While maintaining analytical tractability, our approach incorporates the interaction between seating position, equipment constraints, and COM motion, and focuses on carving‑based gate‑turning dynamics. This balance between simplicity and physical fidelity provides a targeted theoretical basis for adaptive skiing training and underscores the potential of pendulum‑based kinematic frameworks as practical, computation‑efficient tools for performance analysis in disabled alpine skiing.

### Correlation analysis between skiing time and trajectory characteristics

The results of this study indicated a significant negative correlation between single-gate skiing time (${\it\text{t}}_{\text{s}}$) and the minimum distance between the COM and the gate (${\it\text{d}}_{\text{m}\text{i}\text{n}}$) and the lateral distance (${\it\text{d}}_{\text{l}\text{e}\text{v}}$). This finding suggested that short minimum distances and short lateral distances could significantly reduce skiing time, thereby directly corroborating our third hypothesis. These results align with the findings of Supej et al.^5^ and Federolf et al.^26^, highlighting that optimizing the spatial trajectory during gate turns is critical to improving skiing performance.

It should be noted that while the observed correlations strengthen the external validity of our findings, they may also be influenced by potential confounding factors, such as course‑specific gate spacing, snow surface hardness or texture, and the classification of athlete impairment (e.g., type and level of amputation). Variations in these factors could alter the optimal COM–gate distance relationship or affect speed retention, and should therefore be considered in interpreting the correlations and in applying them to different competitive contexts.

Further analysis of the three different turning strategies revealed that turns where the minimum distance occurred after passing the gate resulted in significantly shorter skiing times compared with the other two strategies. This result suggested that pre‑adjusting body posture and carefully planning COM trajectory during gate turns could minimize speed loss to the greatest extent, thereby offering clear support for our second hypothesis that optimizing the interplay between turning radius, skiing speed, and lateral COM distance produces a significant reduction in skiing time. Federolf et al.^27^ emphasized that the relationship between ski edge angle and COM trajectory directly influences turning efficiency. The findings of this study supported this view, demonstrating that the optimization of skiing time relies heavily on the athlete’s precise control over the COM trajectory.

From a sit‑ski‑specific perspective, the effectiveness of each turning strategy is influenced by biomechanical constraints unique to seated skiing—such as reduced or absent active control of the lower limbs, reliance on upper‑body and trunk movements for edging, and the stability limits of the sit‑ski frame and suspension. These constraints can limit the maximum attainable edge angle and affect the timing of COM redirection, thereby shaping the applicability and selection of turning strategies in real‑world training and competition. The findings suggest that coaches should focus on optimizing turning strategies based on the athlete’s specific impairment and equipment setup to maximize speed and minimize time loss during gate turns.

Individual differences in turning trajectories were observed among athletes, including the influence of factors such as gender and amputation classification on turning radius selection and speed maintenance ability. These findings are consistent with the research of Hébert‑Losier et al.^28^, which also highlighted the effect of individual characteristics on alpine skiing performance.

### Balancing turning radius and gate-turning performance

This study found that turning radius has a dual effect on skiing performance: a larger turning radius helps maintain speed but increases skiing distance, while a smaller turning radius may result in greater speed loss. Therefore, athletes must strike a balance between speed and distance to minimize skiing time^29^.

This finding aligns with the research by Gilgien et al.^30^, which stressed that selecting an appropriate turning radius effectively balances speed loss and distance gain, rather than solely pursuing the shortest distance. Particularly in complex gate layouts, optimizing the turning radius is critical to improving overall skiing performance^31^. By using the inverted pendulum model, this study quantified this balance, providing a theoretical basis for technical training. It should be noted, however, that the balance point identified through the model may not be fully generalizable across all athletes, as individual differences in body mass, strength, and technical proficiency can shift the optimal trade-off. Therefore, model calibration at the individual level is recommended for practical application in training.

### Impact of individual athlete differences on skiing trajectories

Although this study did not focus on the specific effects of individual athlete characteristics on skiing performance, data analysis revealed certain differences in COM trajectories and turning strategies among athletes of different genders, classifications, and skill levels. For example, some athletes tended to choose larger turning radii to maintain speed stability, while others opted to shorten their skiing path through more agile posture adjustments before turns. This observation suggested that trajectory optimization not only relies on theoretical models but also requires individualized adjustments based on athlete characteristics.

The findings of Hébert-Losier et al.^28^ and Blauwet et al.^32^ support this observation, showing that para-athletes exhibit highly individualized skiing techniques due to physical conditions and equipment constraints. This study also corroborated these findings, emphasizing that trajectory optimization should incorporate gender, body weight, amputation type, and skill level to design tiered training programs tailored to each athlete.

### Generalizability to non-paralympic populations

Although this study focused on elite Paralympic athletes, the proposed methodology holds significant potential for generalization to non-elite and recreational sit-ski populations. The integrated measurement system and inverted pendulum model could be adapted to assess technical performance across diverse skill levels, from beginners to advanced recreational skiers. For recreational sit-skiers with disabilities, the model could provide valuable feedback on trajectory efficiency and turning technique, potentially accelerating skill acquisition and enhancing skiing safety. The accessibility of drone and IMU technology makes this approach feasible for broader application in adaptive sports programs and rehabilitation settings.

However, key considerations for generalization include: (1) accounting for greater technical variability among non-elite athletes, (2) adapting the model for different sit-ski equipment designs used in recreational settings, and (3) validating the approach across varied terrain conditions encountered by recreational skiers. Future studies should explicitly test this methodology in broader populations to establish its generalizability and develop simplified implementation protocols for non-research settings.

The principles of trajectory optimization identified in this study—particularly the balance between turning radius, speed maintenance, and distance efficiency—likely apply universally across sit-skiing populations, though the specific optimal parameters may vary with individual characteristics and skill levels.

### Limitations

While this study validated the applicability of the inverted pendulum model for sit-ski alpine skiing, several limitations remain.

First, this study treated the athlete and ski equipment as a rigid body system, without fully accounting for the elastic properties of the equipment or the flexibility of the athlete’s body, which may influence the trajectory. While this assumption simplifies the modeling process and reduces the number of parameters, it omits certain biomechanical and mechanical effects that occur in real skiing. For example, the sit-ski frame and ski blades can deform under load, altering effective edge angles and snow contact geometry; likewise, upper-body oscillations and soft-tissue movement can induce subtle shifts in COM position and velocity^33^. Mechanistically, including compliance could modify trajectory-length forecasts in two counteracting ways: (i) equipment flex and soft-tissue motion may introduce small lateral meanders, generally increasing the COM path length; (ii) flex-induced edge angle changes can reduce the instantaneous turning radius, potentially shortening the path. In practice, the first effect (micro-oscillations) often dominates over a full turn, yielding a net longer trajectory and thus reducing the negative bias (underestimation) observed in our rigid-model simulations. Neglecting these factors may lead to underestimation of lateral COM excursions and inaccuracies in the calculation of turning radius and ground reaction forces, especially during aggressive turns or variable snow conditions. Future models could integrate equipment compliance and body-segment flexibility using multi-body dynamics or finite element methods, supported by empirical measurements from strain gauges, high-speed motion capture, or instrumented skis, to improve simulation fidelity and applicability.

Second, the experimental environment was set on a fixed slope and smooth snow surface, without considering the effects of complex terrain, wind speed variations, and snow quality changes typically encountered in real competitions^30^.

Lastly, the sample size was relatively small, involving only 11 elite sit-ski athletes. This limitation was largely due to the restricted availability of high-level athletes within the Paralympic training context. While the observed correlations and effect sizes were moderate to strong, the small sample size inevitably reduces statistical power and may affect the stability of the estimates. Moreover, the homogeneity of the participants — all being elite-level athletes — may limit the generalizability of the findings to broader populations such as developing athletes or those with different impairment classifications. Consequently, the present results should be interpreted with caution.

### Perspective

This study provides important insights into the optimization of sit-ski alpine skiing trajectories using a dynamic modeling approach, bridging a gap left by earlier studies that primarily focused on standing skiing^35^. Building on these findings, several avenues exist to advance both modeling accuracy and practical application.

Future research could enhance the current model by integrating the elastic properties of ski equipment and the flexibility of the athlete’s body through multi-body dynamics or finite element methods^34^, enabling more accurate simulations of real-world skiing dynamics. These improvements could be supported by empirical measurements from strain gauges, high-speed motion capture, or instrumented skis, thereby enhancing simulation fidelity and applicability.

Incorporating environmental variables such as snow quality, slope complexity, wind resistance, and gate geometry, as well as individual characteristics like gender, body weight, and impairment classification, would enable a more refined and personalized understanding of skiing performance^35^. Expanding the testing environment to include dynamic slopes, variable snow conditions, and complex gate layouts would help validate the model’s robustness under diverse competitive scenarios^36^.

In addition, combining this modeling approach with artificial intelligence and machine learning could enable intelligent trajectory optimization and real-time feedback systems for athletes^37^. For example, Tang et al.^38^ developed “SnowMotion,” a wearable sensor-based mobile platform that uses five IMU-based modules (accelerometer, gyroscope, magnetometer) placed on the lower limbs and trunk to capture kinematic data. The data are transmitted via Bluetooth to a mobile app for real-time analysis, 3D motion reconstruction, and digital human visualization, providing actionable technique feedback and training assistance during skiing^37^. Similarly, integrating our modeling framework with such wearable-sensor and AI-driven feedback systems could allow real-time COM tracking and predictive adjustment of turn strategies in training or competition.

Such advancements could have broad implications not only for sports performance optimization but also for sports medicine, aiding in the prevention of injuries and the design of individualized rehabilitation and conditioning protocols tailored to sit-ski athletes. Furthermore, future studies would benefit from increasing both the size and diversity of the participant cohort, potentially through multi-center or international collaborations, to improve statistical robustness and applicability across different performance levels^36^.

## Data Availability

The datasets generated and analyzed during the current study are available from the corresponding author, Xiangdong Wang, upon reasonable request.
